# Editors’ Response to Omotayo: Research Needed on Better Prevention of Pre-Eclampsia

**DOI:** 10.9745/GHSP-D-16-00136

**Published:** 2016-06-20

See related article by Omotayo.

We appreciate points made by Omotayo and colleagues^1^ in their comment on our earlier GHSP editorial on pre-eclampsia and perinatal mortality.^2^ They draw attention to deficiencies in the care of preterm newborns that can limit the impact of other elements of a comprehensive approach to pre-eclampsia. In settings accounting for the greatest burden of mortality attributable to preterm birth, a large proportion of births are in health facilities lacking capabilities for neonatal intensive care. Nevertheless, core elements of good care of preterm and very small newborns can still feasibly be delivered in settings with limited resources.

Kangaroo mother care (KMC) was originally developed in response to limited access to incubators and other elements of neonatal intensive care but was subsequently documented to give better outcomes than conventional high-tech care.^3^ Consequently, KMC is now being promoted in high-income countries due to its effectiveness, not merely as a low-cost alternative. From this experience, it’s evident that much can be done, even in very resource-constrained settings, to improve key elements of care of preterm and very small newborns, notably:

- Good *thermal care*—maximizing skin-to-skin contact
- Conscientious and capable attention given to *feeding*—relying on breastmilk only, to the extent appropriate, and providing any needed support, e.g., breastmilk expression and cup feeding or tube feeding
- Vigilance, through good *nursing care*, to pick up complications early and respond appropriately

Omotayo et al. further elaborate on a point made in the editorial about expected impact from systematic antenatal screening for pre-eclampsia followed by timely delivery. Goldberg and colleagues^4^ have argued persuasively that these factors account for the bulk of the decline in pre-eclampsia–related deaths experienced in high-income countries over the past century.

Their third main argument is that more can be done to prevent pre-eclampsia from developing in the first place, pointing specifically to aspirin and calcium supplementation. Certainly for those at particular risk, notably women with a previous history of pre-eclampsia,^5^ low-dose aspirin is effective. But the proportion of serious outcomes of pre-eclampsia preventable with this intervention is relatively modest. Of greater potential population impact would be widespread uptake of antenatal calcium supplementation.^6^ Available evidence suggests that in populations with low calcium intake, all-cause newborn mortality could be reduced by as much as 30% with near universal coverage of this intervention.

*But* *the problem is the dose of calcium.* The current recommendation from the World Health Organization for antenatal supplementation is 1500–2000 mg (3–4 tablets) of elemental calcium per day. At a weight of 1.25 g/tablet, that translates to a pound and a half of tablets per pregnancy. Furthermore, at this dosing, the cost for antenatal calcium supplementation is close to an order of magnitude greater than for antenatal iron-folate supplementation.

There is reason to believe that a closer-to-physiologic dosing of 500 mg of elemental calcium per day may give similar benefit.^7^ But available evidence is not yet sufficient to warrant changing the current recommendations. In the meantime, this practical problem of weight, bulk, and cost constitutes an important barrier for provision in the low-resource settings where this intervention could give the greatest benefit.

*What is needed is a dose-finding study to determine if a lower dose can reduce risk of serious pre-eclampsia outcomes such as perinatal death to a similar degree to that which has been documented with high-dose supplementation*.

It’s time for one our donor partners (e.g., the United States Agency for International Development, the Bill & Melinda Gates Foundation, or the National Institutes of Health) to step up to the plate to fund the needed research to remove this formidable barrier. –*Global Health: Science and Practice*

The problem: feasibility of delivery of the currently recommended high dose for calcium supplementation.

The solution: a study to determine if low-dose calcium also works.
